# Quantitative assessment of Zirconium-89 labeled cetuximab using PET/CT imaging in patients with advanced head and neck cancer: a theragnostic approach

**DOI:** 10.18632/oncotarget.13910

**Published:** 2016-12-11

**Authors:** Aniek J.G. Even, Olga Hamming-Vrieze, Wouter van Elmpt, Véronique J.L. Winnepenninckx, Jolien Heukelom, Margot E.T. Tesselaar, Wouter V. Vogel, Ann Hoeben, Catharina M.L. Zegers, Daniëlle J. Vugts, Guus A.M.S. van Dongen, Harry Bartelink, Felix M. Mottaghy, Frank Hoebers, Philippe Lambin

**Affiliations:** ^1^ Department of Radiation Oncology (MAASTRO), GROW-School for Oncology and Developmental Biology, Maastricht University Medical Center, Maastricht, The Netherlands; ^2^ Department of Radiation Oncology, The Netherlands Cancer Institute, Amsterdam, The Netherlands; ^3^ Department of Nuclear Medicine, The Netherlands Cancer Institute, Amsterdam, The Netherlands; ^4^ Department of Pathology, Maastricht University Medical Centre, Maastricht, The Netherlands; ^5^ Department of Medical Oncology, The Netherlands Cancer Institute, Amsterdam, The Netherlands; ^6^ Department of Medical Oncology, GROW-School for Oncology and Developmental Biology, Maastricht University Medical Center, Maastricht, The Netherlands; ^7^ Department of Radiology and Nuclear Medicine, VU University Medical Center, Amsterdam, The Netherlands; ^8^ Department of Nuclear Medicine, Maastricht University Medical Center, Maastricht, The Netherlands; ^9^ Department of Nuclear Medicine, University Hospital, RWTH Aachen University, Aachen, Germany

**Keywords:** immuno-PET, Zirconium-89, cetuximab, LAHNSCC, EGFR

## Abstract

Biomarkers predicting treatment response to the monoclonal antibody cetuximab in locally advanced head and neck squamous cell carcinomas (LAHNSCC) are lacking. We hypothesize that tumor accessibility is an important factor in treatment success of the EGFR targeting drug. We quantified uptake of cetuximab labeled with Zirconium-89 (^89^Zr) using PET/CT imaging.

Seventeen patients with stage III-IV LAHNSCC received a loading dose unlabeled cetuximab, followed by 10 mg 54.5±9.6 MBq ^89^Zr-cetuximab. PET/CT images were acquired either 3 and 6 or 4 and 7 days post-injection. ^89^Zr-cetuximab uptake was quantified using standardized uptake value (SUV) and tumor-to-background ratio (TBR), and correlated to EGFR immunohistochemistry. TBR was compared between scan days to determine optimal timing.

Uptake of ^89^Zr-cetuximab varied between patients (day 6-7: SUV_peak_ range 2.5-6.2). TBR increased significantly (49±28%, *p* < 0.01) between first (1.1±0.3) and second scan (1.7±0.6). Between groups with a low and high EGFR expression a significant difference in SUV_mean_ (2.1 *versus* 3.0) and SUV_peak_ (3.2 *versus* 4.7) was found, however, not in TBR. Data is available at www.cancerdata.org (DOI: 10.17195/candat.2016.11.1).

In conclusion, ^89^Zr-cetuximab PET imaging shows large inter-patient variety in LAHNSCC and provides additional information over FDG-PET and EGFR expression. Validation of the predictive value is recommended with scans acquired 6-7 days post-injection.

## INTRODUCTION

Locally-advanced head and neck squamous cell carcinomas (LAHNSCC) are challenging to treat. The majority of patients presents with locally advanced cancers at the time of diagnosis [1]. Although advances in surgery, radiotherapy and systemic therapy have improved survival over the last decade, the prognosis remains poor [2]. Patients with advanced loco-regional disease require multimodality treatment [3]. For (functionally) irresectable tumors, radiotherapy is combined with concurrent cisplatin [4, 5] or with the targeted drug cetuximab [6]. Cetuximab is a human-mouse chimeric monoclonal antibody targeting the epidermal growth factor receptor (EGFR). This receptor activates several pathways that are involved in cell proliferation and survival. The EGF receptor is overexpressed in most LAHNSCC and is related to radio- and chemotherapy resistance [7, 8]. Cetuximab binds to the extracellular domain of EGFR, blocks ligand binding and, as a result, prevents receptor activation [9–12].

Radiotherapy combined with either cisplatin or cetuximab have both shown improved treatment results over radiotherapy alone [6, 13]. However, addition of cetuximab to chemoradiotherapy or substituting radiotherapy combined cisplatin by cetuximab did not show any additional benefit [14–16]. Most likely not all patients will benefit equally from the same treatment, for example due to inter-tumor heterogeneity and patient related factors, making patient tailored treatment essential. Several measures were proposed for predicting cetuximab treatment efficacy, including drug-induced skin-rash, EGFR protein expression and -gene mutations [17, 18]. So far, the predictive value of these markers has been inconclusive. We hypothesize that the accessibility of the cetuximab into the tumor is an important predictive marker in the treatment efficacy [19]. In tumors lacking EGFR expression, response to the targeted drug is unexpected regardless of accessibility, while in tumors with an EGFR overexpression, the accessibility of the tumor is expected to be a determining factor in drug uptake. Imaging with radioactive labeled cetuximab could be used to non-invasively quantify the uptake of cetuximab. Ultimately, drug uptake imaging could be applied in clinic for pre-treatment patient selection (e.g. in combination with decision support systems [20, 21]), and treatment evaluation during therapy.

Since antibodies like cetuximab have a long half-life in the blood pool (69 - 95 hours) radioactive labelling with the long lived positron emitter Zirconium-89 (^89^Zr) was chosen (half-life of 78 hours) [22, 23]. Aerts et al. [19] proved in an animal study that *in vivo* imaging of ^89^Zr-cetuximab is feasible and also showed a disparity between ^89^Zr-cetuximab uptake and EGFR-expression of the tumor cells. Moreover, it was shown in a phase I first in human study that ^89^Zr-cetuximab can be safely administered to patients [24].

The main aims of this study were to quantify the uptake of ^89^Zr-cetuximab in the tumor and involved lymph nodes in patients with LAHNSCC and to determine optimal timing of imaging after ^89^Zr-cetuximab administration. The secondary aim was to correlate ^89^Zr-cetuximab uptake with EGFR expression and metabolic activity as determined by ^18^F-Fluorodeoxyglucose (FDG) PET/CT scan.

## RESULTS

The first 17 patients (12 males, 5 females; age range 45-68y) enrolled in the ARTFORCE study received ^89^Zr-cetuximab imaging and were analyzed. After a minimum follow-up of 2 years, 3 patients presented with a locoregional recurrence and 3 patients developed metastasis. (Supplementary Table 1). Average primary tumor volume was 41.7 ± 24.7 cm^3^. Sixteen of the seventeen patients had regional lymph nodes metastasis. Fifteen patients had ^89^Zr-cetuximab scans at two time points available for analysis; for two patients only the scan at the second time point could be used. One of those patients refused a scan and for the other patient a scan was excluded because the aortic arch was not in the field of view. Those two patients were excluded for the optimal timing and temporal stability analysis; the data was used for the other analyses. All patients underwent pre-treatment FDG PET/CT scan. The patient and tumor characteristics are listed in Table 1.

**Table 1:** Patient characteristics

Patient	Age	Sex	Primary tumor site	Tumor stage			TNM group staging	Primary tumor volume (cm^3^)	HPV status (p16)	EGFR IHC score
				T	N	M				
1	61	M	Oropharynx	T3	N1	M0	III	20	+	270
2	65	M	Oral cavity	T4	N1	M0	IV	54	NA	210
3	55	M	Oropharynx	T4	N2b	M0	IV	53	-	184
4	56	F	Oropharynx	T3	N2b	M0	IV	11	+	207
5	66	F	Oropharynx	T4b	N2b	M0	III	30	-	245
6	45	M	Oral cavity	T4a	N2c	M0	II	98	NA	210
7	62	M	Oropharynx	T3	N3	M0	IV	44	+	80
8	57	M	Oropharynx	T3	N0	M0	III	20	-	235
9	68	M	Hypopharynx	T4	N2b	M0	IV	41	NA	1
10	63	M	Oropharynx	T4	N2c	M0	IV	42	+	10
11	64	M	Oral cavity	T4	N2b	M0	IV	78	NA	70
12	60	F	Oral cavity	T4	N1	M0	IV	21	NA	212
13	50	M	Oral cavity	T4	N2b	M0	IV	76	NA	200
14	55	F	Oropharynx	T4	N1	M0	IV	51	+	180
15	68	F	Oropharynx	T3	N2c	M0	IV	30	+	225
16	55	M	Oropharynx	T3	N2b	M0	IV	10	-	5
17	67	M	Hypopharynx	T3	N2c	M0	IV	29	NA	285

Abbreviations: M = male, F = female, NA = not assessed. HPV status was assessed with p16 immunohistochemistry.

**Figure 1:**
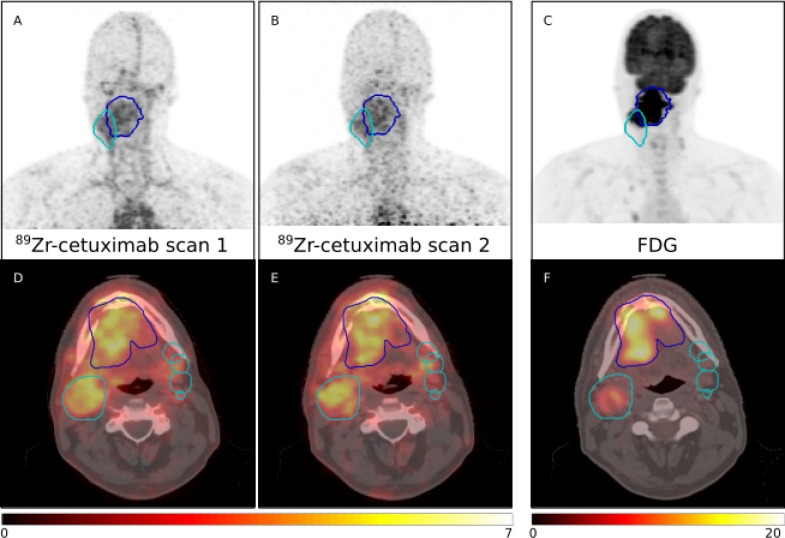
PET maximum intensity projections (MIP) (top row) and fused PET/CT images showing PET uptake in SUV (bottom row) of the two ^89^Zr-cetuximab scans and FDG PET/CT of one patient (patient 6) The GTV for the primary tumor is depicted in blue; the CTV for the lymph nodes in cyan. Only the largest lymph node is displayed in the MIP.

**Figure 2:**
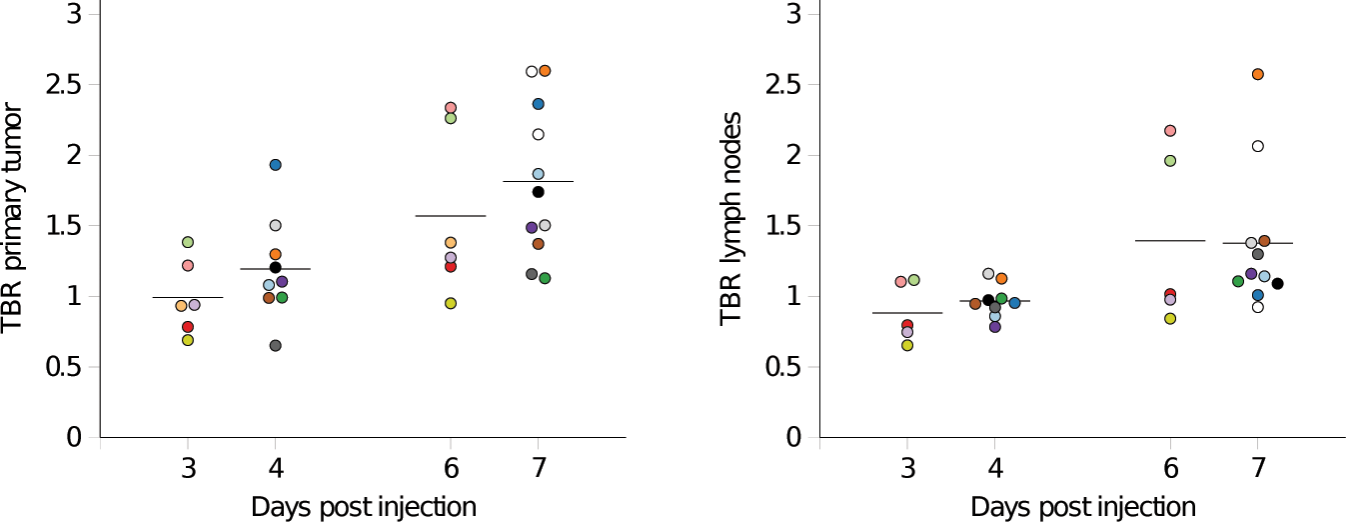
The tumor-to-background ratio (TBR) of ^89^Zr-cetuximab in the primary tumor (left) and maximum TBR in the lymph nodes (right) plotted as a function of the number of days between ^89^Zr-cetuximab administration and PET/CT imaging The bars depict the mean uptake for the individual scan points. Each patient is depicted with a different color. Two patients (white marks) were only scanned on one day.

Quantitative PET analysis showed a large inter-patient variety of tracer uptake. For the first scan the SUV_peak_ ranged between patients from 2.5 - 6.2, SUV_max_ from 2.8 -7.9, SUV_mean_ from 1.8 - 4.0 and TBR from 0.7 - 2.1. For the second scan the SUV_peak_ ranged from 2.5 - 6.2, SUV_max_ from 2.9 - 7.7, SUV_mean_ from 1.6 - 3.9 and TBR from 1.0 - 2.6. Average SUV_peak_, SUV_max_, SUV_mean_ and TBR values for the primary tumor and the lymph nodes, for the first and second ^89^Zr-cetuximab PET/CT scan are shown in Table 2. The ^89^Zr-cetuximab TBR in the primary tumor was for all patients higher on the second scan compared to the first scan. The TBR increased on average with 49% ± 28 % (*p* <0.01), indicating an improved imaging quantification profile at the later time points. The two ^89^Zr-cetuximab scans of an example patient are shown in Figure 1. In Figure 2 the TBR is plotted as function of the number of days after ^89^Zr-cetuximab administration for the individual patients.

**Table 2:** ^89^Zr-cetuximab uptake on scan 1 and 2, the difference of scan 2 compared to scan 1, and FDG PET uptake

		^89^Zr-cetuximab scan 1	^89^Zr-cetuximab scan 2	Difference ^89^Zr-cetuximab (%)	FDG
Primary tumor	SUV_peak_	4.1±1.2	4.0±1.2	−1.3±9.4	14.3±6.9
	SUV_max_	5.0±1.8	4.9±1.6	1.5±12.1	17.6±7.8
	SUV_mean_	2.6±0.7	2.6±0.7	−0.6±11.9	6.4±2.8
	TBR	1.2±0.4	1.7±0.6	49.1±28.1	
Lymph nodes	SUV_peak_	3.4±1.0	3.4±1.2	−7.6±12.9	8.3±5.2
	SUV_max_	4.1±1.2	4.1±1.6	−3.5±12.7	10.9±5.6
	TBR	0.9±0.2	1.4±0.5	43.3±35.0	
Aortic Arch	SUV_mean_	3.6±0.9	2.5±0.9	−31.7±13.4	

**Table 3:** Volumes of high uptake regions of 89Zr-cetuximab and FDG

	^89^Zr-cetuximab scan 1			^89^Zr-cetuximab scan 2		
	^89^Zr-cetuximab TBR > 1.2	^89^Zr-cetuximab TBR > 1.4	FDG > 50% SUV_max_	^89^Zr-cetuximab TBR > 1.2	^89^Zr-cetuximab TBR > 1.4	FDG > 50% SUV_max_
Volume of high uptake region (cm^3^)	20.7±6.4	7.2±6.6	28.4±11.4	21.7±4.7	6.6±4.3	28.3±11.5
DICE	0.4±0.2	0.2±0.2		0.4±0.1	0.2±0.1	

Overlap comparison using the DICE similarity coefficient.

In patients with a high ^89^Zr-cetuximab uptake in the primary tumor, in general also an elevated maximum uptake in the lymph nodes was observed, as shown in Supplementary Figure 1. For the second scan a strong, significant correlation was found between the TBR in the lymph nodes and primary tumor (*r* = 0.76, *p* < 0.01).

The voxel-based comparison between the two ^89^Zr-cetuximab uptake patterns, showed correlation coefficients ranging from 0.18 - 0.86, see Supplementary Table 1. The patients with a low ^89^Zr-cetuximab uptake (TBR < 1.2), and plausible less specific tracer uptake in the tumor, had correlation coefficients of 0.18, 0.20 and 0.66. Excluding these patients with low uptake levels, resulted for the remaining 13 patients in an average spatial correlation of 0.68 ± 0.11 between the two scans.

In Figure 1 (C and F) the FDG PET/CT scan is displayed for comparison with the ^89^Zr-cetuximab PET/CT scan. No correlation was found between the FDG SUV_peak_ and ^89^Zr-cetuximab SUV_peak_ in the primary tumor, for the first (*r* = 0.11, *p* = 0.69) or second ^89^Zr-cetuximab PET/CT scan (*r* = 0.46, *p* = 0.07). Comparison of the high spatial uptake regions showed only minor overlap between high ^89^Zr-cetuximab uptake regions (TBR > 1.2 or 1.4) and high FDG uptake regions (> 50% of SUV_max_). The volumes of the high uptake regions and DICE scores are given in Table 2.

**Figure 3:**
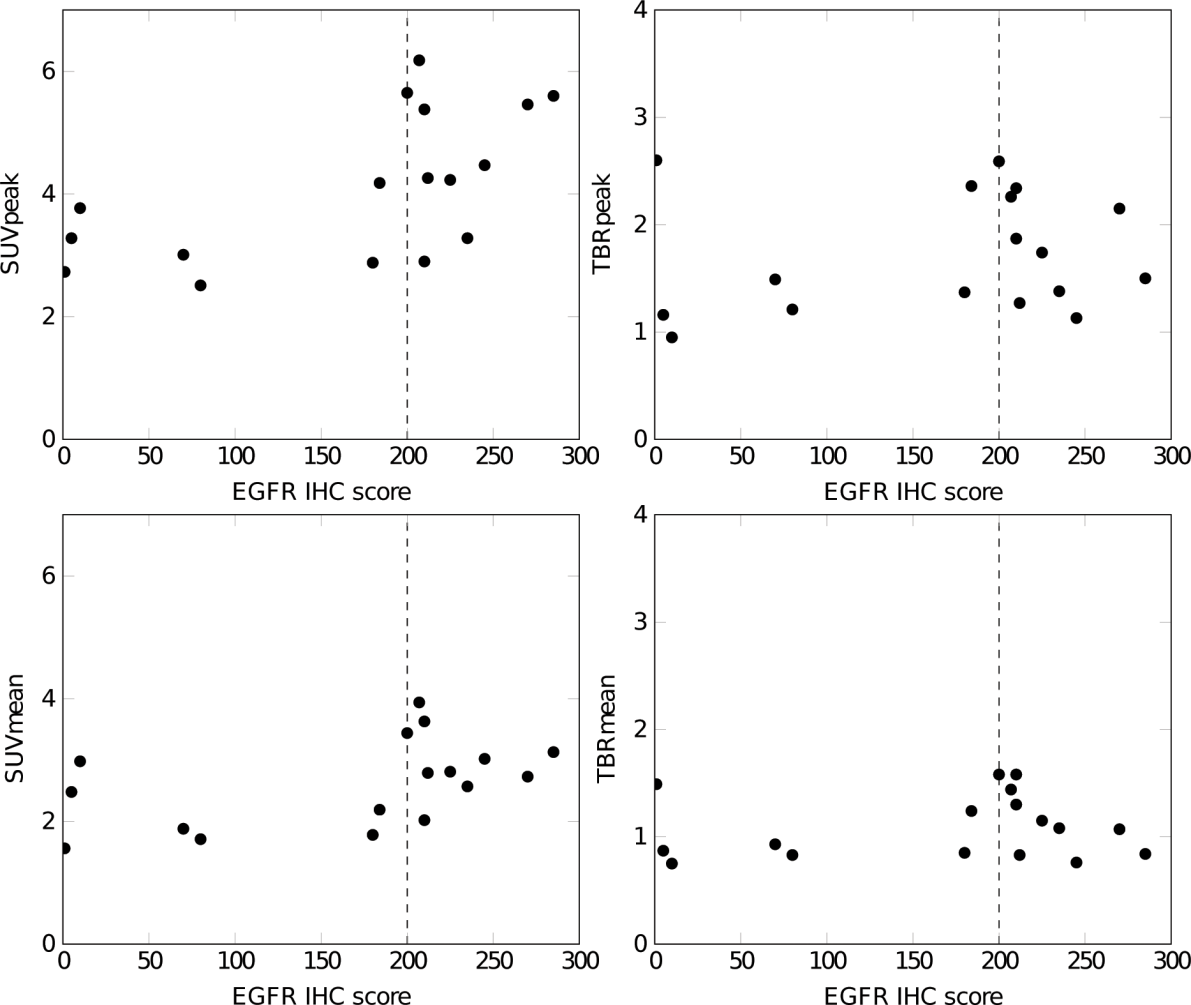
Correlation between the EGFR immunohistochemistry (IHC) score and the ^89^Zr-cetuximab peak and mean TBR, and peak and mean SUV in the primary tumor The PET parameters are calculated for the second ^89^Zr-cetuximab scan (6 or 7 days post-injection). An EGFR IHC score ≥ 200 (dashed line) is classified as high EGFR expression, an IHC score < 200 as low EGFR expression.

The EGFR IHC scores showed seven tumors (41%) with a low EGFR expression, IHC < 200, (IHC: 76±79) and ten tumors (59%) with a high expression, IHC ≥ 200, (IHC: 230±29). Based on the second ^89^Zr-cetuximab PET/CT scan, the SUV_mean_ was 2.1±0.5 and 3.0±0.6 for the low and high EGFR expressing group respectively. The SUV_peak_ was 3.2±0.6 and 4.7±1.1 respectively, the TBR_mean_ 1.0±0.3 and 1.2±0.3, and the TBR_peak_ 1.6±0.6 and 1.8±0.5, where TBR_mean_ and TBR_peak_ are the SUV_mean_ and SUV_peak_ divided by the background uptake. The SUV_mean_ (*p* < 0.01) and SUV_peak_ (*p* < 0.01) were statistically significantly different between the low and high EGFR expression groups, however for the TBR_mean_ (*p* = 0.315) and TBR_peak_ (*p* = 0.417) no statistically significance was observed. In the group with a low EGFR expression, 3 out of 7 (42%) patients had a high ^89^Zr-cetuximab TBR (TBR_peak_ > 1.4); in the group with high EGFR expression 7 out of 10 (70%) patients had high uptake (TBR_peak_ > 1.4). In Figure 3 the PET parameters as a function of EGFR IHC scores are shown.

## DISCUSSION

This study analyzed ^89^Zr-cetuximab PET/CT imaging at two time-points before radiotherapy treatment, to determine the optimal timing of ^89^Zr-cetuximab imaging, the spatial stability of the ^89^Zr-cetuximab uptake patterns, and the uptake in the primary tumor compared to the lymph nodes to allow future use in treatment selection. Furthermore, ^89^Zr-cetuximab uptake was compared with EGFR expression and metabolic activity as determined by FDG PET/CT.

The later ^89^Zr-cetuximab imaging time points were associated with increased tumor to background ratios in all patients, therefore, imaging at 6 or 7 days post-injection is recommended for future studies. This is in agreement with the results in advanced colorectal cancer [25], where also 6 days post-injection was described as optimal imaging time point. The imaging time point did not influence the average peak or maximum uptake; the improvement in TBR between the time points is due to a decrease in background activity. The voxel-based comparison between the two ^89^Zr-cetuximab scans showed that there is a correlation for patients with sufficient uptake of the tracer. The correlation is moderate however, indicating that there is a minor change in spatial uptake patterns over time.

As anticipated, a large variation in ^89^Zr-cetuximab uptake was found between patients. The TBR on the second scan ranged from the background level (TBR around 1.0) to a TBR of 2.6 times the background level. This inter-patient variety could possibly be exploited and used to select tumors that can be targeted by the monoclonal antibody to ultimately predict treatment outcome. However, more research is required to determine which of the calculated measures (SUV_mean_, SUV_peak_, SUV_max_, TBR) best reflects the accessibility of the drug to the tumor. To be able to use ^89^Zr-cetuximab PET/CT imaging for patient selection, the antibody uptake should be related to treatment outcome and an appropriate measure for differentiating the responders and non-responders should be determined. The studied group of patients was unfortunately too small and the received treatments too heterogeneous to link treatment outcome to ^89^Zr-cetuximab uptake. A sample of more than 17 seventeen patients is needed to define such a measure.

The exploratory analysis comparing high uptake regions on the ^89^Zr-cetuximab PET/CT images with the high uptake regions on FDG images revealed only minor overlap (DICE<0.6 for TBR>1.2). A correlation between the two tracers could be hypothesized given that EGFR influences proliferation and thereby glucose metabolism. However, many factors other than EGFR contribute to a difference in metabolism and studies linking proliferation to FDG uptake have shown conflicting results [26, 27].

A significant difference in ^89^Zr-cetuximab SUV was found between the groups with a high and a low EGFR expression. The tumors with a high expression had on average a higher ^89^Zr-cetuximab SUV_mean_ and SUV_peak_. However, when we compared the TBR between the two groups, no significant difference was observed. In tumors lacking EGFR expression, response to the targeted drug was unexpected, while in tumors with an EGFR overexpression, the accessibility of the tumor was hypothesized to be a determining factor in drug uptake. As expected, in the tumors with high EGFR expression a mix of low and high ^89^Zr-cetuximab uptake was observed. Remarkably, high ^89^Zr-cetuximab PET uptake was also observed in the low EGFR expression group; the patient with the lowest EGFR IHC score had the overall highest ^89^Zr-cetuximab TBR. A limitation of this kind of analysis and a possible explanation for this remarkable result is that EGFR expression was only determined for one sample which might not represent the whole tumor [28]. In contrast to a single biopsy, ^89^Zr-cetuximab imaging can give a 3D uptake pattern of the entire tumor. Moreover, the reproducibility of EGFR staining is questionable. Interobserver variability, differences in tissue fixation techniques and increased storage time of the samples could negatively influence reproducibility. For example, Chung et al. [29] found that cetuximab shows activity in tumors that do not express EGFR and concluded that EGFR determined by immunohistochemistry might not reflect the tumor biology.

A high correlation was observed between the ^89^Zr-cetuximab uptake in the primary tumor and the lymph nodes. This might indicate that uptake of the drug is mainly determined by intrinsic characteristics of the tumor cells. Interestingly, the Bonner trial [6] showed in an exploratory subgroup analysis an increased benefit for addition of cetuximab to radiotherapy over radiotherapy alone for patients with nodal involvement, while there was no benefit for patients without nodal involvement. Our data shows that a patient with uptake of cetuximab in the primary tumor will most likely also have cetuximab accumulating in the lymph nodes.

A limitation of this study is that different PET scan settings, e.g. voxel size, slice thickness, scan time and various PET reconstructions algorithms were used in the analyzed patients, complicating quantitative analysis and comparison between patients. After inclusion of the first patients in this study, Makris et al. [30] published work highlighting the importance of harmonization of scan protocols and the suggested scan protocols were adopted. Thereafter, their recommendations regarding image analysis were followed to improve the conformity between scans. For example, scans acquired on a Philips scanner were additionally smoothed and SUV_peak_ was used as measure of tracer uptake since this parameter is less susceptible to noise.

Other factors that could have influenced the imaging results are the cold loading dose and the extra margin for the lymph nodes. A loading dose was used to prevent all labelled antibody going to the liver. As a proof of principle, it was shown before that without a loading dose less than 10% of the injected dose ^89^Zr-cetuximab was available in the blood, while after a loading dose of 500 mg/m^2^ 80% of the injected tracer was detected in the blood and available for tumor targeting [25]. For this study the recommended therapeutic dose of cetuximab (400 mg/m^2^) was used as a loading dose to best represent the clinical situation and to visualize how much cetuximab would reach the tumor during treatment. For the lymph nodes we decided to use the CTV as region of interest. This additional margin, that was added to compensate for small registration uncertainties, would influence the SUV_mean_ of the region. Therefore, only the SUV_max_ and SUV_peak_ were calculated for the lymph nodes. Smaller lymph nodes might suffer from underestimation of the uptake due to partial volume effects.

The ARTFORCE trial was designed to select the most effective treatment, cisplatin or cetuximab, for individual patients. The original design consisted of four treatment arms (two with cisplatin and two with cetuximab) all preceded by an ^89^Zr-cetuximab pre-treatment imaging step which was solely used for research purposes. The use of a long-lived positron emitter complicated procedures for the radiotherapy departments, patients were confronted with an extra radiation burden (0.61 mSv/MBq [31]) and additional guidelines had to be followed by the patient during two weeks after injection to limit radiation exposure to others. The resulting slow accrual in combination with discontinuation of the funding of cetuximab resulted in an amendment of the trial excluding ^89^Zr-cetuximab PET/CT imaging and the cetuximab treatment arms. The imaging results of all patients undergoing the ^89^Zr-cetuximab PET/CT imaging were presented in this study.

In conclusion, the PET tracer ^89^Zr-cetuximab showed a large variation in ^89^Zr-cetuximab tumor-to-background ratio between patients. This inter-patient variety could possibly be exploited and used to select tumors that can be targeted by the monoclonal antibody to ultimately predict treatment outcome. ^89^Zr-cetuximab imaging provides additional information about the accessibility of the drug into the tumor which is not provided by FDG-PET or EGFR expression. Validation of the predictive value is recommended with scans acquired 6 to 7 days post-injection to obtain high tumor to background uptake levels. For future studies a trial design should be chosen that incentivizes patients to participate, e.g. by linking research and therapeutic consequences.

## MATERIALS AND METHODS

### Patient selection and treatment protocol

Patients with previously untreated, histological proven stage III-IV, T3-T4 squamous cell carcinoma of the oropharynx, oral cavity or hypopharynx, were included in the multi-center clinical ARTFORCE trial (ClinicalTrials.gov: NCT01504815). The trial was approved by the appropriate Medical Ethics Review Committee. After giving written informed consent, patients underwent a double randomization: 1. standard radiation dose of 70 Gy or dose-redistribution to the primary tumor to a maximum of 84 Gy on the FDG-avid area and a dose gradient from 70 to 64 Gy in the remainder of the primary tumor. 2. Cisplatin or cetuximab concurrently with radiation. The study protocol is described in detail by Heukelom et al. [32]. For all treatment arms, pre-treatment imaging consisted of one FDG PET/CT scan and two ^89^Zr-cetuximab PET/CT scans. The ^89^Zr-cetuximab PET/CT scans were solely used for research purposes and did not influence any clinical decisions.

### ^18^F-FDG PET/CT image acquisition

Pre-treatment FDG PET/CT scans were acquired at least one day before ^89^Zr-cetuximab administration using the standard clinical protocol following EANM guidelines [33]. All patients were scanned in treatment position: on a flat table top and immobilized using a personalized radiotherapy mask with neck rest and with their arms by their sides.

### ^89^Zr-cetuximab PET/CT image acquisition and analysis

Labelling of cetuximab with Zirconium-89 was performed as described by Verel et al. [34]. Data on the quality of the labelling process can be found in the Supplementary Data. Patients first received an intravenous loading dose of unlabeled cetuximab of 400 mg/m^2^ directly followed by 10 mg ^89^Zirconium labeled cetuximab of 54.5 MBq (range 29 - 62 MBq). ^89^Zr-cetuximab PET/CT images were acquired at 4 and 7 days post-injection (p.i.), corresponding to day -3 and day 1 of radiotherapy, to enable imaging of the therapeutic dose. Alternatively, patients could be scanned on day 3 and 6 p.i. for logistic reasons. If ^89^Zr-cetuximab PET/CT imaging and the first radiotherapy fraction were scheduled on the same day, the PET scan was always acquired before the start of radiotherapy. Patients were scanned in radiotherapy treatment position wearing a personalized radiotherapy mask on either a Philips Gemini TF 16 PET/CT scanner (Philips Healthcare, Best, the Netherlands) or Siemens Biograph TruePoint scanner (Siemens Medical Solutions, Erlangen, Germany). Scans were acquired with a minimum time per bed position of 3 minutes. The Philips PET images were reconstructed using an ordered-subsets time of flight reconstruction technique (BLOB-OS-TF), with 3 iterations and 33 subsets. The Siemens images were reconstructed with a point spread function algorithm (PSF), with either 4 iterations and 14 subsets or 3 iterations and 21 subsets. One scan was reconstructed using the 2D OSEM algorithm with 4 iterations and 8 subsets. All scans were corrected for attenuation, scatter and ^89^Zr decay. Images acquired with the Philips Gemini PET/CT system were additionally smoothed with a Gaussian filter (full width at half maximum of 7 mm) to match the noise levels of the different scanners, as described by Makris et al. [30]. The PET/CT images are publicly available at www.cancerdata.org [37].

### Tumor delineation

Gross tumor volumes of the primary tumor (GTV_prim_) and involved lymph nodes (GTV_ln_) were delineated by an experienced radiation oncologist during the clinical radiation treatment planning process and subsequently propagated to the different scans for further analysis. The delineations were performed either on a dedicated planning CT scan or on the pre-treatment FDG PET/CT scan. In case a dedicated planning CT was acquired, it was first rigidly registered to the CT scan of the FDG PET/CT scan and then the contours were propagated to the FDG PET/CT scan. Thereafter, the CT images of the FDG PET/CT scan were rigidly registered to the CT images of the ^89^Zr-cetuximab PET/CT scans. The tumor delineations were finally copied onto the ^89^Zr-cetuximab scan. All registrations and propagated delineations were visually checked and no registration difficulties were observed. The aortic arch was contoured for assessment of unspecific background uptake of the tracer.

### Quantification of PET tracer uptake

PET/CT images were analyzed using in-house developed Matlab-based software (The MathWorks Inc., Natick, MA). For the FDG PET/CT scan and ^89^Zr-cetuximab PET/CT scans, tracer uptake was quantified using standardized uptake values (SUV) normalized to body weight. The mean uptake (SUV_mean_), maximum uptake (SUV_max_), and peak uptake (SUV_peak_) were assessed inside the delineated tumor sites, where SUV_peak_ is defined as the mean SUV in a 3D sphere with a diameter of 1.2 cm centered at the tumor location with the highest activity. For the primary tumor, the GTV was used as region of interest. For the smaller lymph nodes, the clinical target volume (CTV_ln_) was used as region of interest, which consisted of an isotropic 5 mm extension of the GTV_ln_. SUV_max_ and SUV_peak_ were calculated for the lymph nodes. Furthermore, the average uptake in the aortic arch was calculated and the tumor-to-background ratio (TBR), as defined as SUV_peak_ tumor divided by SUV_mean_ aorta, was determined.

We evaluated which time point after ^89^Zr-cetuximab administration resulted in the largest contrast between tumor and background activity by comparing TBR values for the GTV_prim_ between the first and second scan, to determine the optimal scan time point.

The stability of the ^89^Zr-cetuximab uptake patterns were compared between the two scans. The second scan was registered to the first scan using a rigid registration and the GTV contours of the primary tumors were copied from the planning CT to the first ^89^Zr-cetuximab scan. All registrations were visually checked and no registration problems were observed. A voxel-based correlation between the SUV values of the two scans was calculated.

Finally, ^89^Zr-cetuximab images were compared to FDG PET/CT images. The peak ^89^Zr-cetuximab uptake and peak FDG uptake for the primary tumor were compared. In addition, the location of the high uptake regions on the ^89^Zr-cetuximab and FDG PET/CT scans were compared. For both the first and second ^89^Zr-cetuximab scan, high uptake regions were defined as the volume with a TBR above 1.2 or 1.4. Two cut-off values were used because it is still not well defined which cut-off value qualifies as high uptake. For the FDG PET/CT scans voxels with a SUV above 50% of the SUV_max_ were defined as high uptake region. The overlap between the different volumes was assessed using a DICE similarity score, defined as twice the intersecting volume divided by the sum of both volumes.

### EGFR expression

For all patients a pre-treatment biopsy of the primary tumor was taken, as part of the regular diagnostic examination. Part of the tumor sample was archived in a paraffin block, stored and used for EGFR expression analysis. The archived samples were obtained from the Maastricht Pathology Tissue Collection (MPTC) and NKI-AVL Core Facility Molecular Pathology & Biobanking (CFMPB). Collection, storage and use of tissue and patient data were performed in agreement with the “Code for Proper Secondary Use of Human Tissue in the Netherlands”. The EGFR expression assessment was performed with an EGFR pharmDx qualitative immunohistochemical kit, consisting of two antibodies (Novocastra and Dako, Denmark). All samples were analyzed on the same day in the same lab. EGFR staining intensity was analyzed using a light microscope. The percentages of cells with weak, moderate and strong membranous EGFR staining were scored. An EGFR immunohistochemistry (IHC) score, between 0-300, was calculated according to the formula: EGFR IHC score = 1 x (% cells weak staining) + 2 x (% cells moderate staining) + 3 x (% cells strong staining) [35]. Tumors with an IHC score < 200 were classified as having a low EGFR expression; tumors with an IHC score ≥ 200 as high EGFR expression. This division was based on results of the FLEX study [36]. The EGFR expression was correlated to the ^89^Zr-cetuximab imaging parameters and the EGFR low and EGFR high expression group were compared.

### Statistics

To evaluate the optimal time point for ^89^Zr-cetuximab imaging, the TBR of the primary tumor on the first and second scan were compared using a paired student t-test. Additionally, the Pearson correlation coefficient was used to calculate the correlation between the ^89^Zr-cetuximab TBR in the primary tumor and lymph nodes, to determine the spatial stability between the two ^89^Zr-cetuximab scans, and to determine the correlation between ^89^Zr-cetuximab and FDG peak uptake in the primary tumor. A Mann-Whitney U exact test was used to assess the ^89^Zr-cetuximab parameters between the EGFR high and low uptake groups. Results are presented as mean ± one standard deviation and *p*-values < 0.05 were considered statistically significant.

## SUPPLEMENTARY DATA FIGURE AND TABLES


