# Supplementary material for: Quantitative assessment of Zirconium-89 labeled cetuximab using PET/CT imaging in patients with advanced head and neck cancer: a theragnostic approach
Source: Oncotarget. 2016 Dec 11;8(3):3870–80. doi: 10.18632/oncotarget.13910 (PMC5354801; doi:10.18632/oncotarget.13910)
Supplement: Supplementary file 1 [file oncotarget-08-3870-s001.pdf]

Quantitative assessment of Zirconium-89 labeled cetuximab using PET/CT imaging in patients with advanced head and neck cancer: a theragnostic approach

Supplementary Data

Supplementary Table 1. Imaging characteristics for the ^{89}Zr-cetuximab scans and FDG PET/CT scan for the primary tumor. Treatment characteristics treatment outcome are presented in addition.

	FDG PET/CT			^{89}Zr-cetuximab PET/CT scan 1					^{89}Zr-cetuximab PET/CT scan 2					Correlation ^{89}Zr-cetux scans	Treatment arm	Locoregional recurrence	Metastasis
Patient	SUV_{peak}	SUV_{max}	SUV_{mean}	Day	SUV_{peak}	SUV_{max}	SUV_{mean}	TBR	Day	SUV_{peak}	SUV_{max}	SUV_{mean}	TBR	r			
1	16.37	20.94	7.70	NA	NA	NA	NA	NA	7	5.46	7.73	2.73	2.15	NA	Cetuximab	0	0
2	15.00	17.10	7.75	4	2.67	2.90	2.05	1.08	7	2.90	3.24	2.02	1.87	0.76	Cetuximab	0	0
3	8.61	10.90	4.30	4	4.66	6.82	2.28	1.93	7	4.18	5.71	2.19	2.36	0.53	Cetuximab	0	0
4	12.01	15.20	6.79	3	6.17	7.92	4.01	1.38	6	6.18	7.50	3.94	2.26	0.86	Cisplatin	0	0
5	8.61	9.68	4.66	4	4.09	5.57	2.46	0.99	7	4.47	6.81	3.02	1.13	0.18	Cisplatin	0	0
6	19.14	21.96	8.80	3	5.73	6.52	3.78	1.22	6	5.38	7.03	3.63	2.34	0.60	Cisplatin	0	1
7	8.37	10.87	3.02	3	2.51	2.76	1.82	0.78	6	2.51	2.93	1.71	1.21	0.50	Cisplatin	0	0
8	13.60	16.52	6.56	3	3.17	3.51	2.54	0.93	6	3.28	3.70	2.57	1.38	0.73	Cetuximab	1	0
9	11.85	15.96	5.51	4	3.06	3.41	1.89	1.30	7	2.73	3.23	1.56	2.60	0.68	Cetuximab	1	0
10	11.96	13.22	5.89	3	3.45	3.81	2.70	0.69	6	3.77	4.08	2.98	0.95	0.66	Cetuximab	0	0
11	9.79	13.36	3.01	4	3.72	4.54	1.97	1.10	7	3.01	3.57	1.88	1.49	0.75	Cetuximab	0	0
12	17.34	21.29	9.07	3	4.42	5.15	3.03	0.94	6	4.26	5.19	2.79	1.27	0.78	Cetuximab	1	1
13	37.93	44.17	14.27	NA	NA	NA	NA	NA	7	5.65	6.49	3.44	2.59	NA	Cisplatin	0	1
14	12.24	18.12	2.93	4	3.08	3.66	1.96	0.99	7	2.88	3.74	1.78	1.37	0.61	Cisplatin	0	0
15	16.66	19.87	7.53	4	4.21	4.69	2.94	1.20	7	4.23	4.85	2.81	1.74	0.63	Cisplatin	0	0
16	12.05	14.94	5.16	4	2.83	3.42	1.95	0.65	7	3.28	4.02	2.48	1.16	0.20	Cisplatin	0	0
17	11.53	14.49	6.05	4	6.18	7.56	3.49	1.50	7	5.60	6.58	3.13	1.50	0.80	Cisplatin	0	0

Abbreviations: NA = not acquired. For patient 1 and 3 only the second scan was available for analysis.

Supplementary Table 2. Imaging characteristics for the ^{89}Zr-cetuximab scans and FDG PET/CT scan for the lymph nodes.

	**FDG PET/CT**		**^{89}Zr-cetuximab PET/CT scan 1**				**^{89}Zr-cetuximab PET/CT scan 2**			
Patient	SUV_{peak}	SUV_{max}	Day	SUV_{peak}	SUV_{max}	TBR	Day	SUV_{peak}	SUV_{max}	TBR
1	4.54	8.47	NA	NA	NA	NA	7	2.35	3.74	0.92
2	3.98	7.33	4	2.13	2.26	0.86	7	1.77	1.85	1.14
3	3.68	5.30	4	2.30	3.05	0.95	7	1.78	2.48	1.01
4	18.94	21.38	3	4.98	5.47	1.12	6	5.35	5.67	1.96
5	8.34	10.89	4	4.06	5.57	0.98	7	4.38	6.49	1.11
6	18.59	20.85	3	5.19	6.43	1.10	6	5.01	6.69	2.17
7	5.46	7.14	3	2.55	2.87	0.80	6	2.10	2.42	1.02
8	-	-	-	-	-	-	-	-	-	-
9	9.06	12.59	4	2.65	3.01	1.13	7	2.70	3.44	2.57
10	6.21	7.69	3	3.26	3.92	0.65	6	3.34	3.77	0.84
11	3.52	5.13	4	2.64	3.06	0.78	7	2.35	2.66	1.16
12	7.89	11.40	3	3.51	4.22	0.75	6	3.27	4.04	0.98
13	13.47	17.22	NA	NA	NA	NA	7	4.50	5.24	2.06
14	2.44	3.24	4	2.95	3.42	0.95	7	2.92	3.44	1.39
15	4.57	6.10	4	3.40	3.75	0.97	7	2.65	2.93	1.09
16	10.32	14.06	4	4.01	4.49	0.92	7	3.68	4.31	1.30
17	11.98	15.01	4	4.77	5.65	1.16	7	5.14	6.29	1.38

Abbreviations: NA = not acquired. For patient 1 and 3 only the second scan was available for analysis. Patient 8 did not have involved lymph nodes

Supplementary Table 3. Comparison of the mean uptake in the normal tissues (aorta and trapezius muscle) in the primary tumor for the two ^{89}Zr-cetuximab PET/CT scans. The SUV_{peak} of the tumor is compared to the uptake in the aorta, to calculate the tumour-to-background ratio (TBR), and compared to the uptake in the trapezius, to calculate the tumor-to-muscle ratio (TMR).

	**^{89}Zr-cetuximab PET/CT scan 1**					**^{89}Zr-cetuximab PET/CT scan 2**				
patient	SUV_{mean} aorta	SUV_{mean} trapezius	TBR aorta	TMR trapezius	SUV_{peak} tumor	SUV_{mean} aorta	SUV_{mean} trapezius	TBR aorta	TMR trapezius	SUV_{peak} tumor
1	NA	NA	NA	NA	NA	2.5	0.3	2.1	15.9	5.5
2	2.5	0.4	1.1	6.4	2.7	1.6	0.4	1.9	7.4	2.9
3	2.4	0.3	1.9	15.2	4.7	1.8	0.2	2.4	18.7	4.2
4	4.5	0.4	1.4	13.9	6.2	2.7	0.4	2.3	15.8	6.2
5	4.1	0.6	1.0	7.1	4.1	4.0	0.7	1.1	6.3	4.5
6	4.7	0.4	1.2	14.2	5.7	2.3	0.4	2.3	12.4	5.4
7	3.2	0.3	0.8	8.1	2.5	2.1	0.2	1.2	12.1	2.5
8	3.4	0.3	0.9	9.6	3.2	2.4	0.3	1.4	11.3	3.3
9	2.4	0.3	1.3	10.6	3.1	1.1	0.2	2.6	11.6	2.7
10	5.0	0.4	0.7	9.0	3.4	4.0	0.4	1.0	9.9	3.8
11	3.4	0.4	1.1	9.9	3.7	2.0	0.4	1.5	7.3	3.0
12	4.7	0.6	0.9	7.8	4.4	3.3	0.6	1.3	6.6	4.3
13	NA	NA	NA	NA	NA	2.2	0.2	2.6	25.5	5.7
14	3.1	0.6	1.0	5.1	3.1	2.1	0.5	1.4	5.5	2.9
15	3.5	0.4	1.2	11.6	4.2	2.4	0.4	1.7	11.8	4.2
16	4.3	0.5	0.7	5.9	2.8	2.8	0.5	1.2	6.0	3.3
27	4.1	0.6	1.5	9.9	6.2	3.7	0.8	1.5	6.8	5.6
mean	3.7	0.4	1.1	9.6	4.0	2.5	0.4	1.7	11.2	4.1
stdev	0.9	0.1	0.3	3.1	1.2	0.8	0.2	0.6	5.4	1.2

Abbreviations: SUV = standardized uptake value; TBR = tumor-to-background ratio; TMR = tumor-to-muscle ratio; NA = not acquired.

Labelling of ^{89}Zr-cetuximab

^{89}Zr-cetuximab is produced in compliance with the current Good Manufacturing Practice at the VU University Medical Center. The radiochemical purity was measured by TLC and SEC-HPLC and was 98.4±0.4 % and 98.5±1.4 %, relatively. The mean immunoreactive fraction was 96.6±2.3 %. The filter integrity was on average 3.7±0.5 bar. The edotoxin content was for each batch <0.3 EU/ml.

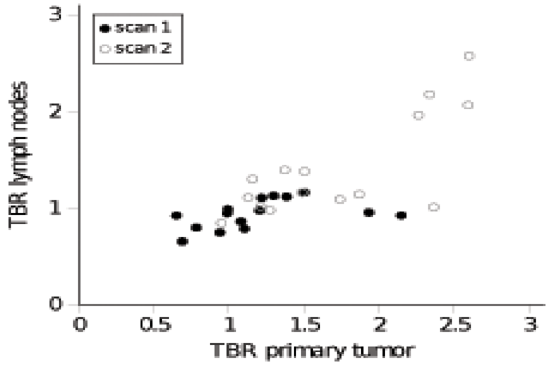

Supplementary Figure 1: Correlation between the ^{89}Zr-cetuximab tumor-to-background ratio in the primary tumor and the maximum TBR of the involved lymph nodes for scan 1 ($r = 0.446$, $p = 0.095$) and scan 2 ($r = 0.764$, $p < 0.01$).
